# The status of nutritional knowledge, attitude and practices associated with complementary feeding in a post‐conflict development phase setting: The case of Acholi sub‐region of Uganda

**DOI:** 10.1002/fsn3.829

**Published:** 2018-10-25

**Authors:** Prossy Nassanga, Ipolto Okello‐Uma, Duncan Ongeng

**Affiliations:** ^1^ Department of Food Science and Postharvest Technology Faculty of Agriculture and Environment Gulu University Gulu Uganda

**Keywords:** attitude and practices, complementary feeding, conflict‐affected communities, knowledge, post‐conflict development situation

## Abstract

Inappropriate complementary feeding is an important challenge to proper child nutrition in post‐conflict rural areas in many sub‐Saharan African countries. While in protected areas during conflict situation and soon after during recovery, communities normally receive nutrition education as part of capacity building to improve knowledge, attitude, and practices to enable them manage maternal and child nutrition issues during the post‐conflict development phase. It is largely unknown whether capacity in nutrition provided is maintained and adequately applied in the post‐conflict development situation. Using Acholi sub‐region of Uganda, an area that experienced violent armed conflict for 20 years (mid‐80s–early 2000), as a case study, we examined the status of nutritional knowledge, attitude, and practices associated with complementary feeding among caregivers of 6‐ to 23‐month‐old children in a post‐conflict development phase following return to normalcy nearly 10 years post‐conflict emergency situation. The results showed that a high proportion of caregivers had good knowledge (88%) and attitude (90.1%) toward complementary feeding. However, only a half (50%) of them practiced correct nutrition behavior. Education status of the household head and sex of the child significantly predicted caregiver knowledge on complementary feeding (*p *≤* *0.05). Education status of the household head also predicted caregiver attitude toward complementary feeding (*p *≤* *0.05). Poverty, food insecurity, and maternal ill health were the major factors that hindered caregivers from practicing good complementary feeding behavior. These results demonstrate that nutrition education on complementary feeding provided to the community during conflict emergency and recovery situation is largely retained in terms of knowledge and attitude but poorly translated into good child feeding practices due to poverty, food insecurity, and maternal ill health. Maternal health, food security, and poverty reduction should be prioritized if adequate complementary feeding is to be achieved among conflict‐affected communities in the post‐conflict development phase.

## 1 INTRODUCTION

Nutrition is a vital component of health promotion and disease prevention (Mowe, Bosaeus, & Højgaard, 2008). The impacts of nutrition on health throughout the course of human life are very profound and are inextricably linked to cognitive and social development, more so in early childhood (Black et al., 2013). The major effects of undernutrition are believed to occur during the first 2 years of human life. This is because, at this stage, undernutrition causes irreversible damage to physical, mental, and social development of the child transcending into reduced intellectual potential at adulthood (Motee, Ramasawmy, Pugo‐gunsam, & Jeewon, 2013; Semahegn, Tesfaye, & Bogale, 2014; WHO, 2010b; World Bank, 2006). Despite the well‐recognized importance of proper child nutrition to health well‐being and human capital development, child undernutrition has remained one of the main public health problems in developing countries (Müller & Krawinkel, 2005; Semahegn et al., 2014). It is one of the most common causes of morbidity and mortality among children in those countries (Amsalu & Tigabu, 2008). It has been estimated that more than 1/3 of the under‐five mortality in developing countries are due to undernutrition‐related diseases (Daelmans et al., 2013; Mesfin, Henry, Girma, & Whiting, 2015). This situation has largely been attributed to inappropriate complementary feeding practices by caregivers who in most cases lack adequate nutrition knowledge and information (Khanal, Sauer, & Zhao, 2013; Shi & Zhang, 2011).

A appropriate diet is critical especially in the first 2 years of a child's life (Rao, Swathi, Unnikrishnan, & Hegde, 2011; World Bank, 2006). This period is considered the most crucial time due to the increased nutritional needs to support rapid growth and development (Semahegn et al., 2014). Infants and young children in developing countries are at an increased risk of undernutrition from 6 months of age onwards when complementary foods are introduced (Memon, Shaikh, Kousar, & Rubina, 2010; Muhimbula & Issa‐zacharia, 2010; Rao et al., 2011). The susceptibility of young children to undernutrition becomes apparent if complementary foods are of low nutrient density and bioavailability (WHO, 2013). On the other hand, if complementary feeding is not carried out properly, it can lead to problems such as diarrhea, growth retardation leading to kwashiorkor, marasmus, and immunodeficiency marked by recurrent and persistent infections which may be fatal (Rao et al., 2011).

Nutritional knowledge, attitude, and practices of caregivers are critical elements that determine the outcomes of complementary feeding regime administered to children (Saha et al., 2008; Turyashemererwa, 2009). It is classically believed that good knowledge should be associated with good attitude and proper nutritional practices (Azizi, Aghaee, Ebrahimi, & Ranjbar, 2011; Mowe et al., 2008). However, in some situations, good knowledge and attitude do not necessarily translate into good practices (Bukusuba, Kikafunda, & Whitehead, 2010). Nonetheless, in order to achieve proper outcome of complementary feeding, it is essential that nutritional knowledge, attitude, and practices should be appropriate. In the context of this study, knowledge is defined as the caregiver's understanding of complementary feeding and nutrition, including the ability to remember and recall food, nutrition and specific pieces of information, and facts essential to complementary feeding. Attitude is defined as the emotional, motivational, perceptive, and cognitive belief that positively or negatively influences the behavior or practice of a caregiver toward complementary feeding. Practice is defined as the observable actions of the caregiver that could affect the nutrition of the child undergoing complementary feeding such as eating, feeding, washing hands, cooking, and selecting foods (FAO, 2014).

In many developing regions of the world, a number of countries have experienced armed conflict (Cortez & Kim, 2012; Gates & Strand, 2012) and many more are currently experiencing the same. Depending on the nature of conflict, the affected communities are either placed in Internally Displaced Peoples (IDP) camps (e.g., Northern Uganda during the 20‐year‐old LRA armed conflict from mid‐80s to mid‐2000) within the country or in Refugee Camps (as is the case with South Sudanese refugees currently in several locations in Uganda). From a nutritional point of view, displaced people living in protected areas are typically confronted with two phases of practical adjustments to cope with. First, being immobilized in protected areas during the emergency situation, mechanisms to ensure proper nutrition especially among vulnerable groups such as women (especially pregnant and lactating) and children (under 5 years) need to be devised. As part of humanitarian assistance during emergency and post‐emergency recovery phase, a number of agencies, especially Non‐Governmental Organizations (NGOs), provide nutrition education and to some extent nutritious food products tailored to nutritional needs of vulnerable people. The second phase of adjustment is during the post‐conflict (or post‐emergency) development phase when conflict‐affected communities return to original homes. In this situation, external humanitarian assistance diminishes and returnees are expected to live economically on their own. Therefore, the returnees should make use of nutrition education provided during the emergency and recovery phases to practice good nutrition behavior.

An important question which has largely remained unanswered is as to whether the capacity building in nutrition provided during emergency and recovery phases of conflict enables the affected communities to exhibit good nutritional knowledge, attitude, and practices during the post‐conflict development phase. This question is important because, during the post‐conflict development phase situation, affected communities are normally confronted with several challenges, and as such, the issue of proper and adequate nutrition of vulnerable people can easily be neglected. Using the Acholi sub‐region of Uganda as a case study, we examined whether, during the post‐conflict development situation, conflict‐affected communities are able to exhibit appropriate nutritional knowledge, attitude, and practices required for a better outcome of complementary feeding of children aged 6–23 months. In Acholi sub‐region, people lived for over a decade in IDP camps from mid‐80s up to mid‐2000 (Saile, Ertl, Neuner, & Catani, 2014) and several humanitarian agencies provided nutritional education to the affected people (ACF (Action Against Hunger), 2004). It is now about 10 years since people in the sub‐region returned to their original villages following official closure of the IDP camps in 2006.

## 2 MATERIALS AND METHODS

### 2.1 Study design, area, and population

A cross‐sectional study design involving in‐depth household interviews and focus group discussions was applied. The study was conducted in Nwoya (02^°^38′N 32^°^00′E) and Amuru (02^°^50′N 33^°^05′E) districts of the Acholi sub‐region. Amuru district is composed of 6 sub‐counties, 32 parishes, and 67 villages while Nwoya district is composed of 5 sub‐counties, 25 parishes, and 63 villages. The two districts were selected because they had the highest rates of undernutrition in the sub‐region (WFP (world Food Programme), 2012) despite substantial nutrition education provided to the community during conflict emergency and recovery situation. The study population consisted of caregivers of children aged 6–23 months old. In the context of this study, a caregiver is the mother of a child or another person who takes care of the child in the absence of the mother. The inclusion criterion was that the caregiver should have received nutritional training while living in the IDP camp and or immediately after camp closure in 2006.

### 2.2 Sample size and sampling framework

The number of participants (*n*) that participated in the study was determined according to the method of Krejcie and Morgan (1970). Using the combined number of households (*N* = 315,678) for Amuru (188,860) and Nwoya (126,818) district (UBOS (Uganda Bureau Of Statistics), 2014), the sample size was determined to be 382 respondents. Household statistics were used to estimate the sample size because data on the number of caregivers are usually unavailable. To locate the study participants, a multi‐stage sampling framework was applied. First, 2 sub‐counties were randomly selected from each district. From the selected sub‐counties, 3 parishes were randomly selected followed by random selection of 2 villages per parish. Random selection of sub‐counties, parishes, and villages was applied because all the two districts were affected by the 20‐year armed conflict. Finally, participants were purposively selected from each village taking into account the inclusion criterion indicated above.

### 2.3 Study instruments

Knowledge, attitude, and practices (KAP) were assessed using a standard questionnaire adapted with modification from WHO (2010a) and FAO (2014). The questionnaire also had a provision for collection of data on socio‐demographic and economic characteristics of the households. With regard to knowledge, questions provided for correct and wrong answers while for practice, caregivers were asked to provide information on how they conducted complementary feeding. For attitude, both positive and negatively framed questions were provided and the respondent had three response options to choose from (disagree, neither agree nor disagree, agree) according to Anand and Puri (2013). A focus group discussion (FGD) guide was used to generate information to supplement what was obtained from household interviews. The instruments were pretested among caregivers of children in the nutrition ward at Gulu Regional Referral Hospital, located in Northern Uganda. This was done to ensure accuracy, clarity, and consistency in the interpretation of questions. After pretesting, responses were analyzed to check for validity and ambiguous questions were rephrased. Another round of pre‐test exercise was conducted in Agwee village, Laroo Division, Gulu district, to check for reliability of the instruments using the Test‐Retest method (Birkman, 2001).

### 2.4 Data collection

Data were collected using research assistants who had previous exposure to nutrition surveys and fluent in English and local language used by all members of the community in the study area (Luo/Acholi). The assistants were conversant with the instruments since they had participated in the pretesting exercise. The assistants were trained only to interpret questions to the study participants but not to assist them in providing answers. Focus group discussions (FGDs) were conducted prior to household interviews. Each FGD constituted of 6–10 members, and a total of 10 FGDs were conducted. This number of FGDs is above the minimum number of six that has been reported to ensure saturation of information in qualitative studies (Guest, Bunce, & Johnson, 2006; Mclafferty, 2004). To ensure originality of information, households that participated in the FGDs were not selected for household interviews. During the interviews with the caregivers, other members of the household were not allowed to participate to avoid influencing the caregiver.

### 2.5 Data analysis

Data from FGDs were transcribed, coded, and common themes established and analyzed using qualitative content analysis method (Cho & Lee, 2014). With regard to knowledge and practices, every correct answer took on a score of 1 while a wrong answer and where the respondent did not know, a score of 0 was given. In the case of attitude, a score of 0, 1, and 2 was given to “disagree,” “neither agree nor disagree,” and “agree” response, respectively. However, reverse scoring was done for negatively framed statements. This meant that a score of 0 was given to “agree” and 2 to “disagree” (Anand & Puri, 2013). Scores on knowledge, attitude, and practices for each respondent were calculated by summing up the scores attained on each question and the overall score ranked as good or poor. Knowledge, attitude, and practice scores were ranked as poor if the overall score fell below 55%, 57.1%, and 62.5%, respectively, according to Ul Haq, Hassali, Shafie, Saleem, and Farooqui (2012). Data on socio‐demographic characteristics were summarized using descriptive statistics (frequency, percentages, mean, and standard deviation). Binary logistic regression was performed to determine socio‐demographic predictors of good knowledge, attitude, and practices, respectively. The logistic regression model used is presented in Equation (1):

(1) $$Y_{i}=\alpha +\beta_{1}X_{1}+\beta_{2}X_{2}+\beta_{3}X_{3}\cdots \cdots \cdots \cdots \beta_{14}X_{14}+\mu$$

where *Y*_*i*_ is the binary dependent variable depicting the status of knowledge, attitude, or practices; *X*_1_ to *X*_14_ are the independent variables; *α* is the regression constant; *β* is the regression coefficient; and μ is the error term. Before running the regression, correlation analysis (Pearson) was run to eliminate highly correlated independent variables (correlation coefficient greater than 0.70) according to Dormann et al. (2013). The following independent variables were selected and used to run the binary logistic regression: sex of the child (male = 1, female = 0), education status of the household head (primary and above = 1, no formal education = 0), sex of the household head (male = 1, female = 0), age of the caregiver (years), marital status of the caregiver (married = 1, otherwise = 0), employment status of the household head (formal = 1, non‐formal = 0), employment status of the caregiver (formal = 1, non‐formal = 0), education status of the household head (primary and above = 1, no formal education = 0), education status of the caregiver (primary and above = 1, no formal education = 0), size of the household, amount of money spent on food per month (UGX), residence of the caregiver (rural = 1, urban = 0), age of the child (months), birthplace of the child (health care center = 1, otherwise = 0), and attendance of nutrition training (yes = 1, no = 0). Binary regression was run separately for each dependent variable. The binary dependent variable was a dummy for good or bad knowledge, attitude, and practices, respectively. Statistical analyses were performed using Statistical Package for Social Sciences (SPSS) version 20.

## 3 RESULTS

Data on socio‐demographic characteristics of the study participants are presented in Table 1. In terms of age, the majority of the caregivers (90%) were between 35 and 45 years, 67.3% of the respondents resided in rural areas, while 84.0% were married. The number of male children in the households sampled was higher than the number of females by 5%. The mean age of the children was 13.8 months and ranged from 6 to 23 months. Majority of them (57.2%) were at 6 months (9.9%), 10 months (7.9), 11 months (9.2%), 12 months (7.9%), 14 months (7.9%), 19 months (7.2%), and 20 months (7.2%). More than ¾ of the infants were born from a health facility. In terms of leadership, the majority (86.6%) of the households sampled were male‐headed. Half of the household heads were involved in farming as the main occupation. Similarly, more than half (61.5%) of the caregivers were involved in farming as the main occupation. Sale of agricultural produce was the main source of income to half of the households where the respondents resided. Besides, households spent a monthly average of Uganda shillings 10, 493 (USD 2.9) on food. In terms of education, the largest proportion of household heads (90.0%) and caregivers (88.2%) had obtained formal education. More than half of the men made decisions on how family income was spent in the household. In most households, women (85.8%) decided on the type of food to be cooked.

**Table 1:** Socio‐demographic characteristics of the respondents in the study area

Variables	*n*	%	Variables	*n*	%
Caregiver's age			Main source of family income		
35–45 years	344	90	Formal employment	40	10.5
46–55	38	10	Casual labor	35	9.2
Residential area			Small‐scale business	66	17.3
Rural	257	67.3	Sale of Agriculture produce	195	51.0
Urban	125	32.7	Small‐scale business & sale of agricultural produces	46	12.0
			Education level of the household head		
Marital status			No formal education	38	10
Single	30	7.9	Primary	164	42.9
Married	321	84.0	Secondary	125	32.7
Separated	26	6.8	Tertiary	55	14.4
Widowed	5	1.3	Education level of the caregiver		
Sex of the child			No formal education	45	11.8
Male	201	52.6	Primary	255	66.7
Female	181	47.4	Secondary	64	16.8
Source of child's birth			Tertiary	18	4.7
Hospital	342	89.5	Decision on expenditure of family income		
Home	40	10.5	Husband	263	68.8
Sex of the household head			Wife	66	17.3
Male	331	86.6	Husband & wife	27	7.1
Female	51	13.4	Mother in‐law	13	3.4
Occupation of the household head			Father‐in‐law	10	2.6
Not employed	32	8.4	Grand parents	3	0.8
Formal employment	51	13.4	Decision on the food cooked in the household		
Small‐scale trading	47	12.3	Husband	40	10.5
Casual labor	43	11.2	Wife	313	81.9
Farmer	209	54.7	Mother‐in‐law	15	3.9
Occupation of the caregiver			Family	14	3.7
Not employed/Housewife	43	11.2			
Formal employment	13	3.4			
Small‐scale trading	66	17.3			
Casual labor	22	5.8			
Farmer	235	61.5			
Others	3	0.8			

Data based on sample size of 382 caregivers. A caregiver is the mother of a child or another person who takes care of the child in the absence of the mother.

Information on complementary feeding situation in the community generated from FGDs is presented in Table 2. There were contradicting views with regard to the adequacy of complementary feeding in the community, initiation of complementary feeding at the recommended time, and the recommended feeding frequency. Some respondents argued that complementary feeding practices in the community were adequate while others disagreed. A number of local foods were used by the community in complementary feeding. When asked about awareness of commercial complementary foods, only a few of the community members were aware while most of them were not. The community cited packaged soy, soy mixed with silverfish (*Rastrineobola argentea*), biscuits, commercial soft drinks (e.g., soda) among others as typical examples of commercial complementary foods known to them. However, the community reported that these foods were expensive for them and that they needed a local complementary food formula. Despite the existence of many sources of information on complementary feeding which were reported as being beneficial, the community needed more information especially on formulation of nutritious complementary foods, advantages of good nutrition among other aspects. The community also appreciated the fact that inappropriate complementary feeding negatively affected the nutritional status of their children as poorly fed children were malnourished and weak. A number of challenges which affect complementary feeding were reported and these concentrated around poverty, food insecurity, and health complications such as swollen breasts for mothers and HIV/AIDS. However, when asked about how the complementary feeding situation in the community can be improved, the community proposed introducing a local complementary food formula, increasing the accessibility of commercial complementary foods especially to the local markets so that people who are able to buy can have access to them.

**Table 2:** Community perspective on complementary feeding situation

Aspects of complementary feeding discussed	Community views/perspective^a^
Adequacy of complementary feeding	Generally, the community believes that it is adequate because caregivers should follow advice from the hospital. However, adequacy depends on the household and specific mothers. Some household lack food while some mothers may have health complications which bar them from following the recommended practices
Initiation of complementary feeding at 6 months	Varies from household to household. Majority of the caregivers start at 6 months while others start early (3 or 4 months) due to various health complications including inadequate breast milk and/or swollen breasts/breast engorgement
Feeding at the recommended meal frequency (2–3 and 3–4 times for 6‐ to 8‐month‐old and 9‐ to 23‐month‐old children, respectively)	Some caregivers adhere while others do not. Most caregivers do not adhere because of too much work/responsibilities at home. Following the recommended meal frequency is also challenged by weather changes which affect crop yields and availability of food
Foods commonly given to children 6–23 months	Porridge from millet (*Eleusine coracana*) or maize (Zea mays) flour, pasted green vegetable, for example, pasted cowpea (Vigna Unguiculanta*)* leaves and spider plant (Chlorophytum comosum), bean (Phaseolus vulgaris) soup, silver fish (*Rastrineobola argentea*), eggplant (Solanum melongena), cabbage (Brassica oleracea, sweet potato (Ipomea batatas), potato (Solanum tuberosum), millet bread, soups obtained from the family's food, eggs (in a few families), salty water mixed with groundnut (Arachishypogaea), or sesame (*Sesamumindicum L*.) paste
Awareness about complementary foods on the market	A few caregivers are aware but the majority lack information
Examples of commercial complementary foods known to the community	Packed soy (*Glycine max*), soy mixed with silverfish and millet, maize flour mixed with soy, packed milk, millet mixed with silverfish, glucose and corn flakes, biscuits, soft drinks (e.g., soda, juice)
Affordability of commercial complementary formulae	Largely unaffordable
Availability of locally adapted complementary food formulae	Largely unavailable
The need for locally adapted formulae in the community	The community expressed need for training on formulating nutritious complementary foods using locally available food resources
Source of information on complementary feeding	Hospitals, friends, women groups, grandmothers, mothers‐in‐law, village health teams, relatives, radios, own instinct from birth, and Non‐Governmental Organizations.
Benefit and adequacy of information on complementary feeding from the sources named	For a few households, the information has helped to keep children in a healthy state and prevent some of the nutrition‐related diseases. But for most of the households, the information is inadequate
Other aspects of complementary feeding that the community would be interested to receive information on	Formulation and preparation of nutritious infant foods, hygiene, estimation of the right quantity for infant feeding, education about advantages of good nutrition, and the recommended meal frequency
Perception about the effect of inappropriate complementary feeding on the nutritional status of children	Caregivers appreciated that inappropriate complementary feeding is bad because some children in the community who are poorly fed become very weak and malnourished.
Challenges experienced by mothers in the community to implement good complementary feeding practices	Main challenges included: lack of financial resources to procure nutritious foods for the children, thus most households feed children on the same food type for over a week (lack of dietary diversity); inability to breastfeed due to pain in the breasts/swollen breasts/engorgement; Changing weather pattern which affects the yields of different crops; HIV/AIDS; and limited availability of diverse food categories
Suggestions to improve complementary feeding	Increase accessibility of affordable complementary foods, trainings on formulation of nutritious foods from locally available food resources, need for a local formula for producing infant feeds, and training on aspects of sanitation, nutrition, and agriculture

Information provided by caregivers of children 6–23 months during Focus Group Discussions (FGDs); 10 FGDs were conducted. A caregiver is the mother of a child or another person who takes care of the child in the absence of the mother.

**Figure 1:**
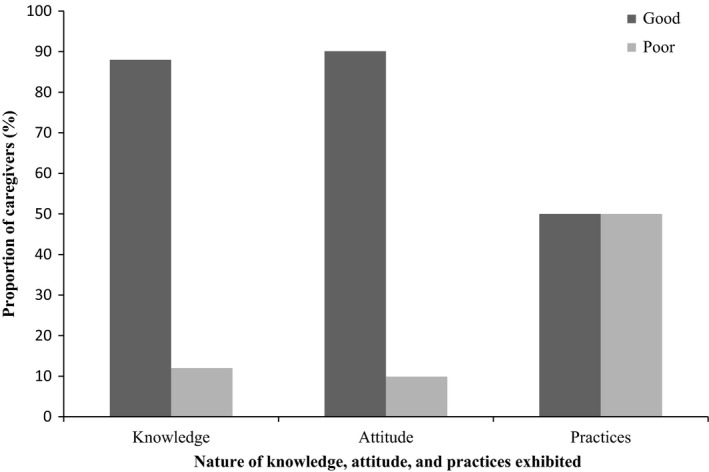
Distribution of knowledge, attitude, and practices on complementary feeding among caregivers. Data based on a sample size of 382 caregivers. A caregiver is the mother of a child or another person who takes care of the child in the absence of the mother

The distribution of scores on knowledge, attitude, and practices regarding complementary feeding among caregivers is presented in Figure 1. With regard to knowledge and attitude, the proportion of caregivers who had good nutrition knowledge and good attitude toward complementary feeding was above 80%. On the other hand, the proportion of caregivers with good practices on complementary feeding was identical to those who exhibited poor practices (Figure 1).

Data on specific aspects used to test caregiver's knowledge on complementary feeding are presented in Table 3. The proportion of caregivers with correct responses was higher (above 60%) than those with wrong responses for all aspects tested with the exception on the aspect testing the importance of including animal foods in a child's diet.

**Table 3:** Proportion of caregivers who provided correct responses to specific aspects testing knowledge on complementary feeding

Aspect of knowledge on complementary feeding tested	Proportion of caregivers that gave correct answers	
	(*n*)	(%)
Importance of early breastfeeding	231	60.5
How often to breastfeed	326	85.3
Breastfeeding duration	325	85.1
Appropriate age for introducing complementary food	329	86.1
Nature of a complementary food	347	90.8
Reasons for introducing complementary foods at 6 months	291	76.2
Risks of late complementary feeding	253	66.2
Risks of early complementary feeding	266	69.6
Minimum meal frequency for 6‐ to 8‐month children	304	79.6
Minimum meal frequency for 9‐ to 11‐month children	307	80.4
Minimum meal frequency for non‐breastfeeding children	251	65.7
Minimum dietary diversity	274	71.7
Importance of including animal foods in a child's diet	140	36.6
Exclusive breastfeeding	278	72.8

Data based on sample size of 382 caregivers. A caregiver is the mother of a child or another person who takes care of the child in the absence of the mother.

Data on specific aspects of complementary feeding used to test attitude are presented in Table 4. Generally, caregivers’ attitude toward complementary feeding was good and above 70% with the exception that more than half of them found breastfeeding for 2 or more years embarrassing.

**Table 4:** Proportion of caregivers who agreed to the aspects used to test attitude toward complementary feeding

Specific aspects of complementary feeding tested	Proportion of caregivers who agreed to the aspects tested	
	*n*	%
It is important to breastfeed a child within one hour after birth	342	89.5
It is important to breastfeed a child on demand	322	84.3
I do not find breastfeeding for 2 or more years embarrassing^a^	164	42.9
It is good to give a child of less than 2 years other foods other than breast milk^a^	296	77.5
I am so mindful about the quality of food that I give to my baby (texture, nutritional composition)	347	90.8
I think introducing food late could affect my child	320	83.8
I am disturbed when I introduce complementary food to a child of less than 6 months^a^	309	80.9
It is important for my child to consume different types of foods	342	89.5
It is helpful to include animal foods in the child's diet	347	90.8

Data based on sample size of 382 caregivers. A caregiver is the mother of a child or another person who takes care of the child in the absence of the mother.

a The question was reverse coded.

Distribution of caregivers’ adherence to recommended complementary feeding practices is presented in Table 5. Ninety percent of the respondents adhered to continued breastfeeding along with complementary foods. However, less than 50% of the respondents gave their children the right consistency (6 months old: thick mashed or semi‐solid food; 8 months old: finger foods; and 12 months old: family food) of complementary foods, met the minimum meal frequency (2–3 and 3–4 times per day for breastfed children 6–8 and 9–23 months, respectively, and 3–4 times for non‐breastfed 6‐ to 23‐month‐old children), introduced complementary foods at 6 months of age and ensured dietary diversity (consumption of food from at least 4 or more food groups).

**Table 5:** Distribution of caregivers’ adherence to recommended complementary feeding practices

Recommended complementary feeding practices	Proportion of caregivers that adhered	
	(*n*)	(%)
Continued breastfeeding along with complementary foods	344	90.1
Nature of food/food consistency for 6‐ to 8‐month‐old, 9‐ to 11‐month‐old, and 12‐ to 23‐month‐old children	113	29.6
Minimum meal frequency for 6–8, 9–11 months and non‐breastfed children	105	27.5
Introduction of complementary foods		
At 6 months	134	35.1
Before 6 months	196	51.3
After 6 months	52	13.6
Ensuring dietary diversity (children who received foods from 4 or more food groups)	181	47.4

Data based sample size of 382 caregivers. A caregiver is the mother of a child or another person who takes care of the child in the absence of the mother.

Results of binary logistic regression analysis used to test the effect of socio‐demographic factors on knowledge, attitude, and practices regarding complementary feeding are presented in Table 6. Only, the educational status of the household head and the sex of the child were significant predictors of knowledge on complementary feeding (*p* = 0.009 and 0.005, respectively). The relationship is presented in equation (2).

**Table 6:** Socio‐demographic predictors of good knowledge, attitude, or practices on complementary feeding among caregivers

Socio‐demographic characteristics	Knowledge			Attitude			Practices		
	β	*SE*	*p*‐Value	β	*SE*	*p*‐Value	β	*SE*	*p*‐Value
Sex of the household head	1.181	1.735	0.496	16.519	6396.436	0.998	0.618	1.064	0.561
Age of the caregiver	0.082	0.081	0.311	−0.108	0.067	0.107	0.029	0.036	0.422
Marital status	−1.597	1.588	0.315	16.097	6188.859	0.998	−0.363	0.981	0.711
Employment status of the household head	18.418	10214.364	0.999	0.333	1.634	0.839	−0.821	0.869	0.345
Employment status of the caregiver	−0.244	1.459	0.867	−1.401	1.279	0.273	0.295	0.765	0.700
Education status of the household head	−2.870	1.094	**0.009**	−3.199	1.257	**0.011**	−0.896	0.680	0.188
Education status of the caregiver	0.402	1.192	0.736	1.305	1.150	0.257	−0.251	0.607	0.679
Size of the household	0.228	0.161	0.156	−0.014	0.189	0.942	0.027	0.084	0.750
Amount of money spent on food per month	0.000	0.000	0.935	0.000	0.000	0.687	0.000	0.000	0.523
Residence of the caregiver	0.524	0.819	0.522	0.721	0.947	0.447	−0.652	0.407	0.109
Sex of the child	2.764	0.973	**0.005**	−0.607	0.784	0.439	−0.083	0.357	0.816
Age of the child	0.048	0.072	0.506	−0.146	0.080	0.067	−0.066	0.037	0.076
Birthplace of the child	−1.224	0.943	0.194	0.655	1.140	0.566	−0.653	0.613	0.287
Attainment of nutritional training	0.328	0.707	0.643	1.369	0.917	0.135	−0.634	0.382	0.097
Constant	−21.309	8732.47	0.998	−11.675	9445.6	0.999	1.223	1.215	0.314

*β*: regression coefficients. *SE*: standard error. A caregiver is the mother of a child or another person who takes care of the child in the absence of the mother.

(2) $$Y=-21.309-2.870X_{1}+2.764X_{2}$$

Where *Y* represents good knowledge on complementary feeding, *X*_1_ is the dummy for the educational status of the household head (primary and above = 1; and no formal education = 0), and *X*_2_ is the dummy for sex of the child (male = 1 and female = 0). By equation (2), an increase in the education level of the household head by one unit reduces the level of nutritional knowledge of the caregiver by a factor of 2.9. On the other hand, having a male child is associated with a 2.8‐fold increase in the level of caregiver's nutritional knowledge. With regard to attitude, educational status of the household head was the only significant predictor (*p* = 0.011) (Table 6). The prediction is presented in equation (3).

(3) $$Y=-11.675-3.199X_{1}$$

where *Y* represents good attitude towards complementary feeding and *X*_1_ is the dummy for the educational status of the household head (primary and above = 1; and no formal education = 0). According to equation (3), an increase in the educational level of the household head by one unit is associated with a 3.2‐fold decrease in the status of the attitude of the caregiver toward complementary feeding. However, none of the socio‐demographic factors significantly predicted good complementary feeding practices (*p* > 0.05).

## 4 DISCUSSION

Nutrition education is an important vehicle for achieving good nutrition and nutrition‐related health outcomes (Negash et al., 2014). When adequately provided, nutrition education should enable the recipient to attain reasonably good nutrition knowledge, have a positive attitude toward good nutrition, and above all exhibit good nutritional practices (Ickes et al., 2017; Inayati et al., 2012; Vardanjani, Reisi, Javadzade, Pour, & Tavassoli, 2015). If other factors are favorable, it would be expected that a combination of good nutritional knowledge and positive attitude toward nutrition should be appropriately translated to different aspects of nutritional practices/food habits (Rezaee, Azizi, & Hoseini, 2012). Much of the understanding gained on the impact of nutrition education on nutritional knowledge, attitude, and practices has been derived from studies conducted among communities living in peaceful environments. However, little is known about how nutrition education provided to communities living under stressful conditions (e.g., refugee camps) impact on their nutritional knowledge, attitude, and practices when they return to peaceful conditions. This study therefore examined the lacuna with a specific focus on complementary nutrition of children 6‐23 months using the Acholi sub‐region of Uganda as a case study.

Interestingly, the results presented in Figure 1 revealed that whereas a high proportion of caregivers had good knowledge and attitude regarding complementary feeding, most of them had poor nutritional practices. This implies that nutrition education capacity building provided to caregivers during armed conflict emergency situation and immediately during the post‐conflict recovery period is largely retained in terms of knowledge and attitude but limitedly translated into practice. The inability of caregivers to translate good nutritional knowledge and attitude to good nutrition behavior is in consonant with the generally known phenomenon that good nutritional knowledge and attitude may not necessarily guarantee good nutritional practices (Anand & Puri, 2013; Das & Mukherjee, 2014; Hasnain, Majrooh, & Anjum, 2013; Salarkia, Amini, Abdollahi, Eshrati, & Neyestani, 2014), thus suggesting that other factors could be directly responsible or playing a moderating role. Nevertheless, none of the socio‐demographic factors investigated in this study could predict good nutritional practices (Table 5). This is in contrast with findings from other studies which show that maternal/caregiver education (Kumar, Arora, Midha, & Gupta, 2015; Adnan & Muniandy, 2012; Olwedo, Mworozi, Bachou, & Orach, 2008; Tessema, Belachew, & Ersino, 2013) and employment (Ogunba, 2015; Taddele, Abebe, & Fentahun, 2014) as key predictors of good complementary feeding practices, albeit under different contextual situations.

Education is an important factor which determines the adoption and use of recommended health practices (Mokori & Orikushaba, 2012; Vardanjani et al., 2015). The low level of formal education attained by the respondents in the study area (Table 1) could partly be responsible for the low adherence to recommended complementary feeding practices observed (Figure 1). A closer look at Table 1 reveals that most of the caregivers (67.1%) had attained only primary education. Education affects employability potential (Wambugu, 2011). People who are unemployed find it difficult to afford some of the basic necessities and requirements for their children. Poverty and food insecurity might also account for the poor status of complementary feeding practices. This was clearly revealed by the caregivers during FGDs (Table 2). Poverty has been reported in many other areas as a hindrance to complementary feeding. Victor, Baines, Agho, and Dibley (2014) attested to this in a report which showed that poor economic status was one of the main risk factors for inappropriate complementary feeding practices in Tanzania.

Dietary diversity is essential for achieving good nutrition outcome from complementary feeding (Moursi et al., 2008). Table 5 shows that only a small proportion (47.4%) of the caregivers adhered to the recommended dietary diversity level. Agriculture is the principal economic activity in Acholi sub‐region of Uganda. FGD exercise revealed that changing weather pattern led to poor crop yields, and food insecurity and poverty in turn (Table 2). In a broader sense therefore, limited adherence to the recommended dietary diversity in complementary feeding can in part be attributed to food insecurity and poverty occasioned by the inability of households to adapt to weather changes. The dependence of food and nutrition security on agriculture in rural areas in developing countries has long been recognized (Titus & Adetokunbo, 2007). Classically, food and nutrition security can be met through the market (after the sale of produce) and/or own production pathways (Baiphethi & Jacobs, 2009). However, as demonstrated in this study, crop failure as reported by the community during FGDs (Table 2) is a critical factor that requires attention at both policy and household practice levels.

Despite the fact that a high proportion of caregivers (80%) had good nutritional knowledge, there were certain critical aspects where they exhibited deficiency. For instance, very low proportion of caregivers were knowledgeable about the importance of including animal foods in the child's diet (Table 3). Whereas this situation could in part be attributed to the low level of education among the caregivers (Table 1), it is also possible that caregivers might not have received adequate information on this specific aspect of nutrition knowledge. This suggests that the various sources of information on complementary feeding revealed by caregivers during FGD (Table 2) may not be providing sufficient information on such a critical aspect. On the other hand, based on results presented in Table 2, many of the food types used as complementary food were plant‐based and rarely of animal origin, while commercial complementary food formulae were largely unaffordable. This finding justifies the need to build the capacity of rural households to develop and apply complementary food formulae based on locally available food resources as demanded by the caregivers during FGDs (Table 2). In doing so, it would be important, however, to recognize nutrient bioavailability limitations associated with plant‐based foods due to the presence of antinutritional factors (Doss, Pugalenthi, Vadivel, Subhashini, & Anitha, 2011; Mugendi, Njagi, Kuria, Mwasaru, & Mureithi, 2010). Therefore, efforts aimed at making available community adaptable complementary food formulae should incorporate easily adaptable strategies for improving nutrient bioavailability.

Education status of the household head and sex of the child were the major predictors of good nutritional knowledge (Table 6). Based on the coefficient of the child sex element in equation (2), it is apparent that caregiver's knowledge on complementary feeding is 2.8 times better when the baby is a boy child compared to when it is a girl child. This is not surprising because in many developing countries, especially in rural settings, the boy child is always more treasured compared to the girl child in terms of resource allocation (Dercon & Singh, 2011; Mehtabul & Geeta, 2011). This observation draws credence from studies conducted in Ethiopia which showed that mothers who had baby boys timely initiated complementary feeding than those who had baby girls (Semahegn et al., 2014). The negative effect of the presence of a girl child on nutritional knowledge of the caregiver can compromise the quality of complementary feeding regime offered the girl child and nutritional outcomes as well. Strategic efforts are required to deal with the identified child sex disparity in complementary feeding. With respect to education status element of equation (2), it is interesting to note that an increase in education status of the household head is associated with a 2.9‐fold decrease in caregiver's knowledge on complementary feeding. Education status of the household head also affected caregiver's attitude, but negatively. Looking at the coefficient of equation (3), it is evident that an increase in the education level of the household head is associated with a 3.2‐fold decrease in attitude toward complementary feeding. Education is associated with employability, a factor which affects the length of time of interaction between the educated household heads and the caregivers (Wambugu, 2011). In the context of this study, it can be asserted that educated household heads are easily employed and spend most of their time at work, and as such, they are unable to adequately share knowledge on complementary feeding with the caregivers. Therefore, since the caregivers are unable to receive adequate information from the household heads, their attitude toward complementary feeding is negatively affected in the long run. A major limitation of this study is that the community in the study area could have received some nutrition education which influenced the results. Future studies should control for this scenario.

## 5 CONCLUSIONS

The results of this study have demonstrated that nutrition education on complementary feeding provided to the community during conflict emergency and recovery situation is largely retained in terms of knowledge and attitude but poorly translated into good complementary feeding behavior in the post‐conflict development phase. Poverty, food insecurity and maternal ill health are the major factors that hinder care giver's efforts to adhere to recommended (good) complementary feeding practices. Maternal health, food security improvement, and poverty reduction should be prioritized if good complementary feeding is to be achieved among conflict‐affected communities in the post‐conflict development phase.

## ACKNOWLEDGMENT

The authors are grateful to the Regional Universities Forum for Capacity Building in Agriculture (RUFORUM) for funding the study under grant numbers RU 2014 NG 15, RU 2014 NG 13, and RU 2014 GRG‐098.

## CONFLICT OF INTEREST

The authors declare no conflict of interest.

## ETHICAL REVIEW

The study was approved by the Gulu University Research Ethics Committee (GUREC/02/07/2016). Participation in the study was voluntary. Therefore, full consent of participants was sought and each participant signed a consent form before engaging in the study. Participants were assured of confidentiality of information provided, and codes were used for personal information such as names and location to ensure anonymity.
